# The hedgehog pathway in hematopoiesis and hematological malignancy

**DOI:** 10.3389/fonc.2022.960943

**Published:** 2022-08-25

**Authors:** Tucker Lemos, Akil Merchant

**Affiliations:** Samuel Oschin Comprehensive Cancer Institute, Cedars-Sinai Medical Center, Los Angeles, CA, United States

**Keywords:** hematological malignancy, glasdegib, smoothened inhibition, Gli3, GLI1, acute myeloid leukemia, hedgehog (Hh), hematopoiesis

## Abstract

The Hedgehog (HH) pathway is a promising therapeutic target in hematological malignancies. Activation of the pathway has been tied to greater chances of relapse and poorer outcomes in several hematological malignancies and inhibiting the pathway has improved outcomes in several clinical trials. One inhibitor targeting the pathway *via* the protein Smoothened (SMO), glasdegib, has been approved by the FDA for use with a low dose cytarabine regiment in some high-risk acute myeloid leukemia patients (AML). If further clinical trials in glasdegib produce positive results, there may soon be more general use of HH inhibitors in the treatment of hematological malignancies.While there is clinical evidence that HH inhibitors may improve outcomes and help prevent relapse, a full understanding of any mechanism of action remains elusive. The bulk of AML cells exhibit primary resistance to SMO inhibition (SMOi), leading some to hypothesize that that clinical activity of SMOi is mediated through modulation of self-renewal and chemoresistance in rare cancer stem cells (CSC). Direct evidence that CSC are being targeted in patients by SMOi has proven difficult to produce, and here we present data to support the alternative hypothesis that suggests the clinical benefit observed with SMOi is being mediated through stromal cells in the tumor microenvironment.This paper’s aims are to review the history of the HH pathway in hematopoiesis and hematological malignancy, to highlight the pre-clinical and clinical evidence for its use a therapeutic target, and to explore the evidence for stromal activation of the pathway acting to protect CSCs and enable self-renewal of AML and other diseases. Finally, we highlight gaps in the current data and present hypotheses for new research directions.

## Introduction

The Hedgehog pathway was originally described by Nüsslein-Volhard and Wieschaus in reference to a mutant drosophila phenotype that produced a spiky embryo (1). It is one of several critical embryonic body patterning pathways and its role in development has been extensively studied and characterized since its original discovery. Its expression is limited in most healthy adult tissues.

The oncological relevance of the pathway was independently realized by Kinzler et al. when they discovered a gene that had a fifty-fold amplification in some human gliomas (2). It was later discovered that this Glioma-associated oncogene family (GLI1, GLI2, and GLI3) was the mammalian homolog for the HH-responsive transcription factor cubitus interuptus (Ci) (3, 4).

The link between aberrant activation of the pathway and tumorigenesis is not limited to gliomas; it has been implicated in many cancers including basal cell carcinoma, breast cancer, gastric cancer, pancreatic cancer, and various hematological malignancies (5–7). This paper will be limited to the last of these, but for a review of the HH pathway in cancer generally see Skoda et al., 2018 and Scales and de Sauvage 2009 (8, 9).

## Canonical hedgehog signaling

**Figure 1:**
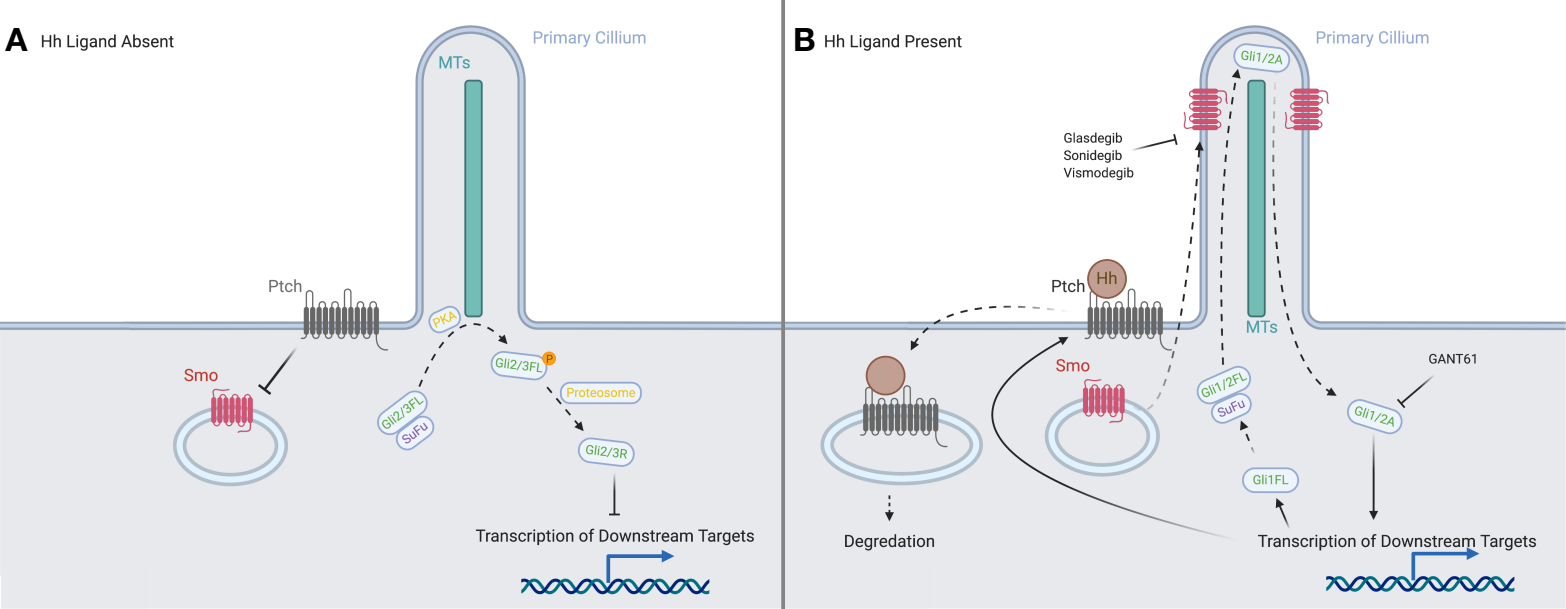
A representation of the canonical HH signaling in the presence and absence of a HH ligand. **(A)** With the ligands absent, PTCH prevents SMO from translocating to the primary cilium. In the absence of SMO, the full-length forms of GLI2 and GLI3 are phosphorylated by PKA, and then processed into their repressor forms, which inhibit downstream transcription. **(B)** When ligands are present, they are bound by PTCH and both are internalized and degraded. This allows the translocation of SMO to the tip of the primary cilium, where it processes the full-length forms of the GLI proteins into their activator forms, which then promote transcription of downstream targets in the nucleus. Created in BioRender.

A simplified schema of the activation of the Hedgehog signaling pathway (see **Figure 1**) principally focuses on four families of proteins: the Hedgehog ligands, the transmembrane protein Patched (PTCH), the transmembrane protein Smoothened (SMO), and the Gli family of transcription factors (Gli1, Gli2, Gli3) (8).

There are three different HH ligands, Indian hedgehog (IHH) which is expressed in early hematopoietic tissues (10) and has a role in modulating chondrocyte development (11), Desert hedgehog (DHH) which regulates development of the peripheral nerves (12) and is essential to spermatogenesis (13), and Sonic hedgehog (SHH). SHH is the most widely expressed and best studied of the HH ligands, being involved in embryonic body patterning.

In the absence of the ligand, PTCH is localized to the base of the primary cilium (PC), where it inhibits SMO activity, likely by preventing SMO modification *via* cholesterol (14, 15). We have shown that human blood and bone marrow cells have primary cilia and mediate hedgehog signaling (16). The canonical activation of the signaling cascade is caused by the binding of a HH ligand to PTCH (17, 18) upon which both are internalized, and SMO moves from vesicles in the cytosol to the PC (19–21), joining the other members of the pathway: the GLI family and the machinery responsible for processing it (22). The translocation of SMO modulates the post-translational processing of GLI2 and GLI3 in the PC: allowing GLI2 to act as an activator and preventing GLI3 from being processed into its strong repressor form (23, 24). Another protein, Suppressor of Fused (SUFU), which normally acts to inhibit GLI activators and sequester them in the cytosol (25–27), allows them to travel to the nucleus, where they upregulate PTCH and GLI1 (28), creating both positive and negative feedback loops to tightly control the downstream signal. The activator forms of GLI1 and GLI2 set off a signaling cascade, reaching downstream targets such as MYCN (29), BCL-2 (30) and VEGF-A (31), many of which are associated with oncogenesis.

Further complexity in transducing this signal comes from its significant overlap and crosstalk with other developmental pathways, especially WNT and NOTCH (32).

## Modes of HH signaling

**Figure 2:**
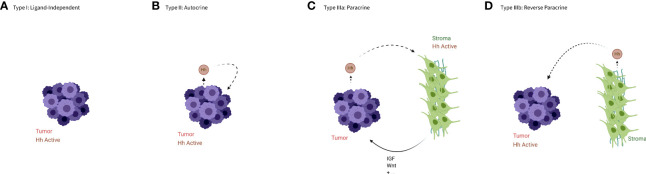
An illustration of the types of aberrant HH signaling. **(A)** In type I signaling, the Hh pathway is active despite the absence of the Hh ligand. **(B)** In type II or autocrine signaling tumor cells both produce and bind the ligands. **(C)** In type IIIa or paracrine signaling tumor cells produce ligands that activate HH signaling in the stroma, which in turn produces a more favorable niche for the tumor cells or cancer stem cells. **(D)** In type IIIb or reverse paracrine signaling the stroma produce ligands to activate HH signaling in the tumor cells. Created in BioRender.

The HH pathway has been shown to regulate proliferation, apoptosis, and angiogenesis, therefore it is not surprising that activation of HH signaling is a common factor in many cancers. Signaling in this context can be broken up into four types, based on the activity of the Hh ligand (see **Figure 2**), though these types are not necessarily mutually exclusive.

### Type I –ligand independent

Type I signaling occurs when the pathway is constitutively active without regard to the presence or absence of a HH ligand. This is often caused by loss of function mutations in the PTCH gene that produce a protein unable to prevent SMO from activating the pathway, as first demonstrated by patients with nevoid basal cell carcinoma syndrome, or Gorlin syndrome (33). These individuals carry an ineffective copy of the PTCH gene and are at an elevated risk of developing various tumors, especially basal cell carcinomas (BCCs) (34, 35). Alternatively, activating mutations in SMO are often found in spontaneous BCC, and rarely in other tumor types (36–38).

Constitutional activation can also be effected by mutations to other elements of the signaling pathway, such as gain of function mutations in SMO that counteract inhibition by PTCH demonstrated in sporadic BCCs (36), loss of the GLI3 suppressor *via* methylation in AML (39) and inactivation of SUFU in medulloblastoma (40).

### Type II – autocrine/juxtacrine

Type II signaling occurs when the pathway is activated by ligands that are produced by the tumor cell itself, or by other nearby tumor cells. This self-targeted increase in HH expression has been observed in a variety of tumors, including but not limited to those of the digestive tract, brain, and lung (41–45).

### Type IIIa – paracrine

Type III signaling occurs when the pathway is activated by ligands produced by other, functionally distinct cells. Type IIIa refers specifically to tumor cells producing ligands to induce HH signaling in non-malignant cells. This was first demonstrated in epithelial tumor cells that produced HH ligands but relied on the pathway being activated in nearby stromal cells rather than binding the ligands themselves (46–48). The stromal cells, upon binding HH, upregulate other signaling factors such as insulin-like growth factor (IGF) and Wnt (46), which can stimulate tumor growth both directly and by creating a tumor promoting microenvironment.

Type IIIa signaling has also been demonstrated to act in a tumor-suppressing manner in some solid cancers, further complicating our understanding of this pathway (49).

### Type IIIb – reverse paracrine

Type IIIb signaling refers to paracrine signaling that occurs in a reverse manner, with stromal cells stimulating tumor growth through secretion of HH ligands, which activate the pathway in tumor cells. This signaling pattern has been demonstrated most clearly in B-cell malignancies (50, 51) and multiple myeloma (52). Though this mode of signaling has been almost exclusively tied to hematological malignancies, there is evidence of stroma-produced HH ligands in some gliomas (53).

### Signaling in cancer stem cells

Hedgehog signaling in cancer stem cells is of particular interest and may employ any of the modes discussed above. CSCs have been implicated as an important part of understanding the response of various cancers to therapy. CSCs are a subset of slowly dividing cells that exist within a tumor that can proliferate, differentiate and reconstitute a heterogenous tumor (54). They are resistant to conventional chemotherapy due to their relatively slow growth and have been consistently implicated as a cause of relapse in many difficult to treat tumors. CSCs have been shown to be supported and maintained by HH signaling in a variety of tumors, such as CML (55), MM (52) and AML (56, 57). In some cases autocrine signaling between differentiated tumor cells and cancer stem cells has been demonstrated while in other cases paracrine signaling between cancer stem cells and the tumor microenvironment has been implicated. For a thorough review of the role of HH signaling in CSCs, see Cochrane et al., 2015 (54).

## A possible requirement for stromal HH signaling in normal and malignant hematopoiesis

### Conflicting data for the role of HH in normal hematopoiesis

Before exploring the HH pathway in hematologic malignancies it is helpful to understand what is known about the HH signaling in normal hematopoiesis.

Early studies showed increased hematopoietic stem cell (HSC) proliferation when SHH was added *in vitro* (58). A role for HH signaling in HSC differentiation was further supported by studies in zebrafish models (59).

The strongest evidence of the HH pathway’s role in HSC renewal comes from mouse models. Trowbridge et. al., found that mice hemizygous for Ptch (increasing HH pathway activity) had significantly increased numbers of HSC progenitor cells and, further, the prolonged HH signaling could exhaust the ability of HSCs to self-renew (60). Our group has shown that loss of Gli1 transcription factor is associated with decreased cell cycling of HSC and delayed recovery of myelopoiesis after radiation or chemotherapy. These mice had no loss of steady state bone marrow chimerism in transplantation experiments, demonstrating that Gli1 is likely only required for the burst of proliferation required during stress hematopoiesis (61).

While studies of Ptch and Gli1 suggest an important role of HH signaling in hematopoiesis, gene deletion studies of Smo, the central hub of hedgehog signaling, have been less clear. Dierks et al. reported that mice fetal liver cells lacking one or both copies of Smo were unable to retain their *in vitro* colony-forming potential in replating experiments, while cells hemizygous for Ptch were far more successful than the wildtype in forming colonies after being replated (62). In transplantation experiments, they showed that loss of Smo delayed engraftment kinetics, however, long-term, steady-state hematopoiesis does not require Smo, as no difference was observed between the number of HSCs, B-cells, or other cell types in mice transplanted with Smo knockout fetal liver HSCs (62). In contrast, Zhao et al. used conditional Smo knockout mice crossed with Vav-Cre mice, which results in conditional knockout of a Smo in the HSC compartment, to show that mice with Smo deficient HSCs had significantly lower rates of blood reconstitution after transplantation, demonstrating a requirement of Smo for HSC renewal (55). Hofmann et al. and Gao et al. both independently found that, using the same conditional Smo allele, this time crossed with an inducible Mx-1-Cre strain, Smo was dispensable for normal hematopoietic function (63, 64). Studies with human HSCs found no reduction in differentiation capacity with inhibiting Smo, both *in vitro* with human HSCs and *in vivo* with the hematopoietic compartments of mice transplanted with HSCs from human cord blood (65).

We have hypothesized that these seemingly contradictory results may be due to different patterns of Cre expression in Vav-Cre and Mx-1-Cre models (66). While both systems are active in the primitive stem cell compartment, Vav-cre shows additional activity in bone marrow stroma and endothelial precursors. An in-depth review of the various HSC-targeting Cre systems can be found in Joseph et al., 2016 (67). The differences in the two models are demonstrated in the similarly difficult issue of determining the role of Wnt/β-catenin signaling in HSCs, where the Vav-Cre model also indicated a requirement for β-catenin for normal HSC function and the Mx-1-Cre model revealed that it was dispensable (68). Therefore, a HSC extrinsic requirement for Hedgehog and Wnt signaling pathways in bone marrow stroma, but not intrinsic to HSCs themselves could explain this discrepancy.

### Evidence for stromal signaling

We propose that, rather than targeting the bulk tumor, glasdegib and other Smo inhibitors are instead acting on stromal cells that promote tumor growth and reconstitution through maintenance of a hospitable niche.

In preclinical models, Smo inhibitors are not directly cytotoxic, but seem to reduce proliferation and self-renewal (55, 60, 69). This is borne out in clinical trials, where the addition of glasdegib to chemotherapy did not improve CR rates. Rather, the effects of glasdegib appear to increase the overall survival (OS) of patients by reducing relapse. One plausible mechanism for this observation is that glasdegib is targeting the leukemic stem cells that mediate relapse. However, direct evidence that SMO inhibitors act on CSC in a cell autonomous fashion are lacking. Meanwhile, evidence from animal vav-Cre models discussed above suggest that loss of Smo in the stroma leads to a loss of HSC self-renewal. This suggests the possibility that Smo inhibitors could act on the bone marrow stroma and thus modulate CSC self-renewal.

Indeed, evidence for such a mechanism can be found in studies on the interaction of HH and retinoid signaling in multiple myeloma. In an elegant series of studies Ghiaur et al. were able to show that stromal production of the enzyme CYP26 can inactivate retinoic acid signaling, preventing differentiation and functionally expanding HSC self-renewal capacity (70). They further demonstrated that stromal production of CYP26 dependent on Smo activity. In a follow up study using a mouse model there were able to show that stroma-specific knockout of Smo sensitizes otherwise refractory multiple myeloma to bortezomib (71).

## Preclinical data supporting the HH pathway as a therapeutic target in hematologic malignancies

As HH is a common driver of tumorigenesis and chemo-resistance, inhibition of the pathway presents a clear target for therapy. HH components have been found to be aberrantly activated in a variety of hematological malignancies, and further, can often be implicated as markers of a poor prognosis in patients, in AML (57, 69) and in CML (65). In this section we summarize the preclinical studies in several tumor types that have demonstrated potential clinical applications of HH inhibitors.

### Chronic myeloid leukemia

Chronic myeloid leukemia (CML) is a hematological malignancy that results when HSCs acquire the fusion oncogene BCR-ABL, leading to constitutive activation of the ABL tyrosine kinase. The advent of tyrosine kinase inhibitor (TKI) therapy has dramatically improved the prognosis of this disease; the treatment is able to consistently effect cytogenic and molecular level responses in early chronic-phase patients (72). CML is often used as an exemplar of a CSC-driven cancer and until recently TKI therapy was considered a lifelong requirement due to CSC-driven reconstitution of the disease upon the end of the treatment (73, 74). Although, recent evidence and practice has moved towards ending TKI therapy in select patients who have achieved deep-molecular responses, only half of these patients maintain their response after discontinuing TKI therapy and many never achieve the deep responses needed to attempt discontinuation (75). Therefore, for these patients or those who develop TKI resistant mutations, further treatment options are needed. One of these candidates has been HH inhibitors, as the pathway has been shown to be aberrantly activated in CML patients. There is increased expression of the pathway in progenitor cells, which becomes more pronounced as the disease progresses (55, 62, 65).

*In vitro* and *in vivo* experiments have returned promising results with HH inhibitors, with treatments reducing the proliferative and self-renewal capacity of CML cells.

Dierks et al. used a viral transgenic model of BCR-ABL in murine bone marrow to show that treatment with cyclopamine, a SMO inhibitor, caused a significant decrease in colony-forming potential and a reduction of the percentage of BCL-ABL+ myeloid progenitors (62). Using the same model, Zhao et al. demonstrate that cells with elevated levels of Numb, a cell determinant that is upregulated in SMO knockout cells, have reduced colony-forming potential. They show that treatment with a SMO inhibitor can reduce the colony number in murine models, in primary human CML, and in the imatinib-resistant T315I mutant of the disease (55).

Glasdegib, vismodegib and sonidegib, three SMO inhibitors, have all been found to significantly decreased progenitors in samples from blast crisis patients and chronic phase patients, while having no significant effect on the colony-forming potential of normal cord blood samples (65, 69). SMOi effect against CML is thought to be mediated, it part, through preventing hedgehog mediated quiescence of cancer stem cells. Transduction of progenitors with an inactive version of GLI2 led to abrogated cell-cycle dormancy, indicating that the pathway may be integral to maintaining this mechanism of chemoresistance (65).

In mice models, downregulation of the pathway through either inhibition or knockout of Smo increases survival, even in some TKI-resistant forms of the disease. Meanwhile, constitutive activation of Smo *via* the SmoM2 mutant allele, survival time significantly decreased when compared to control mice (55, 76). Transplant experiments with BCR-ABL+ Smo -/- fetal liver cells have shown that Smo was required for reconstitution of CML CSCs and thereby of the disease (62). Smo inhibition *via* either glasdegib or sonidegib, combined with TKI therapy drastically reduces engraftment and preventing serial transplantation, implying that Smo inhibition could potentially be combined with TKI to enhance their therapeutic activity (65, 69).

Taken together, these studies have formed a rationale for trials combining SMO inhibitors with TKI therapy, both as a treatment option in rare forms of TKI-resistant forms of the disease and to increase rates of complete eradication of the disease.

### Acute myeloid leukemia

Acute myeloid leukemia (AML) has been one of the most promising targets of HH inhibition therapy, either with Smo inhibitors or with further downstream inhibitors of GLI1 and GLI2 activators.

High level activation of HH signaling is found in a subset of AML patients, is associated with progression of myelodysplastic syndrome (MDS) to AML, and with reduced rates of overall survival of AML patients (57, 77–79). The SMOi glasdegib achieved FDA approval based on a Phase 2 clinical trial and a review of pre-clinical data can help illuminate potential mechanisms of action for this observed benefit. Potential therapeutic mechanisms of HH inhibitors include down-regulation of pro-survival or apoptotic pathways, loss of CSC dormancy, or modulation of chemo-resistance.

The most straightforward mechanism is the pathway’s ability to regulate cell survival and apoptosis through downstream targets such as AKT. HL-60/RX, a radiation-resistant form of the AML model HL-60 cell-line, had elevated expression of GLI-1 and SMO compared to normal HL-60 cells (80). Inhibition of the pathway using a Smo inhibitor (sonidegib), sensitized the HL-60/RX to radiation. In addition, inhibiting Smo reduced expression of elements in the PI-3K/AKT pathway, downregulating apoptosis (81) and has been linked to aberrant activation of HH signaling in other cancers (82). These results are supported by RNA-seq data, which shows that relapsed/refractory AML patients are associated with higher expression of *GLI1* and *PI3K (* 56 *).*

Another area where malignant HH signaling could enhance tumor chemoresistance is through the maintenance of malignant progenitor populations in dormancy. As chemotherapy is cell cycle selective, the slowed division of CSCs allows them to survive treatment, after which they can reconstitute the disease (83). Direct evidence connecting this effect to HH signaling is evident in AML, where cells treated with SMO inhibition (glasdegib) showed significantly fewer dormant CD45+ cells and were sensitized to Ara-C treatment (84).

HH signaling can also effect drug resistance to both cytotoxic and targeted chemotherapy *via* glucuronidation of the therapeutic molecules. Zahreddine et al., 2014, observed increased GLI1 levels upon relapse of patients (85). Other studies have found that patients with increased evidence of HH activity showed greater resistance to Ara-C and to the anti-viral drug ribavirin, which is being clinically evaluated as a therapy targeting the oncogenic eukaryotic translation initiation factor eIF4E (86). In GLI1-elevated patients, there was a decrease of ribvarin-bound eIF4E that could be reversed upon Smo inhibition. Further, GLI1 knockdown reduced levels of UGT1A, a drug- glucuronidating enzyme, and mass-spec analysis revealed that both Ara-C and ribavirin were glucuronidated in drug-resistant cells. Both the resistance and glucuronidation were reversed upon SMOi (85). In stabilizing UGT1A, GLI1 activation can be directly tied to drug-resistance *via* glucuronidation.

The FLT3 internal tandem duplication mutation (FLT3-ITD) appears in 25% of cases of AML and has a significant negative prognostic impact (87, 88). FLT3-ITD patient samples and cell lines have increased expression of GLI2, and FLT3-ITD transgenic mice show rapid progression from myeloproliferation to acute leukemia when crossed with SmoM2 transgenic mice that have constitutive HH activation (89). These data suggest that FLT3 and HH signaling can cooperate to drive leukemic progression, although the precise mechanisms of interaction between these pathways have not been worked out (89).

Complicating matters further, several recent papers have shown varied mechanisms of SMO-*independent* upregulation of GLI activators, all of which are inherently resistant to SMO inhibition. GLI1 upregulation has been shown to be integrated with PI3K independent of SMO (90). We have reported on SMO-independent activation of the pathway caused by loss of *GLI3* expression associated with hypermethylation of the *GLI3* locus. This leads to loss of GLI3R transcriptional repression and unopposed HH target activation through GLI1 and GLI2 (39). Further work has found that primary AML cells from some patients with high GLI1 expression will have reduced proliferation and self-renewal capacity when treated with the GLI1 and GLI2 inhibitor GANT61 but not when treated with SMOi (57). These data suggest that SMOi alone may not be adequate to target HH activation in AML and that direct GLI inhibitors may be required.

### Chronic lymphocytic leukemia and acute lymphoblastic leukemia

While myeloid malignancies have been the main target of investigation for HH inhibition therapies, there is also preclinical evidence that patients diagnosed with lymphoid leukemias may also find clinical benefit in inhibition of this pathway.

As in AML and CML, both acute and chronic lymphoid leukemias seem to respond to HH inhibitors. In B-cell ALL, cancer stem cells that were treated with a Smo inhibitor showed decreased self-renewal potential (91), a result mirrored by the subset of GLI1 rich T-cells when they were treated with either a GLI or SMO inhibitor (92). In T-ALL specifically, there is evidence that inactivating mutations of PTCH speed progression of NOTCH-induced disease, and restoration of WT PTCH activity can induce apoptosis in PTCH mutant T-ALL cells *in vitro*.

In B-cell chronic lymphoblastic leukemia (CLL), HH activation increases proliferation and resistance of CLL cells and is associated with progression of the disease (93, 94). As in other malignancies, GLI1 upregulation is tied to a worse prognosis and predicts response of cells to HH inhibition (95, 96). Inhibition of HH signaling *via* SMOi can sensitize GLI1 positive B-CLL cells to chemotherapy and induce higher rates of apoptosis (95). However, many cases show primary resistance to SMOi (96), in which case a response requires alternative therapies, such as targeting downstream of SMO with GANT61 (93, 94, 97).

As in AML, finding different ways to target the HH pathway should be considered to properly account for tumors resistant to SMO inhibition. This could be in the form of new Smo inhibitors that can be effective against resistant mutants of the protein or in pursuing direct inhibitors of GLI activators such as GANT61.

## Clinical data

The wealth of preclinical data on normal and aberrant HH signaling has guided our interpretation of clinical trials of HH inhibitors. The first FDA-approved HH inhibitor was the SMO inhibitor GDC-0449 (vismodegib, Genentech) for use in relapsed or advanced basal cell carcinomas (BCCs) in 2012 (109). Approval of vismodegib has since been followed by FDA approval of LDE-225 (sonidegib, Novartis) for advanced BCCs in 2015 (110).

Within the scope of hematological malignancies, PF-04449913 (glasdegib, Pfizer) is currently the only FDA-approved HH inhibitor. In 2018, glasdegib was approved for use in newly diagnosed cases of acute myeloid leukemia in combination with low dose cytarabine (LDAC) for patients that are not candidates for intensive induction chemotherapy (111).

Several SMO inhibitors have been tested in clinical trials for hematological malignancies, as summarized in **Table 1**. Vismodegib clinical trials have been run in myelofibrosis, multiple myeloma, and select lymphatic malignancies, but none of them demonstrated the efficacy required to encourage further study. An early phase 1 trial of Sonidegib combined with AZA in various myeloid malignancies, (NCT02129101) did not see decreased remission rates (108). However, especially in AML patients, the trial found increases in both OS and rates of SD. A later phase II trial was stopped early due to lack of efficacy (NCT01826214). Two other SMO inhibitors IPI-926 (Sardegib) and BMS-833923 were studied in myelofibrosis and CML, respectively. Sardegib treatment of myelofibrosis patients did show modest clinical activity, however it failed to reach pre-specified endpoints for clinical efficacy. Specifically, several patients had reductions in spleen size and/or GLI1 levels in the BM. (NCT01371617) (106). BMS-833923 was added to dasatinib in CML with the intention of reducing self-renewal capacity of CML stem cells. The study showed some efficacy, with 3 out of 10 dasatinib-resistant chronic phase CML patients demonstrating clinical benefit with one patient achieving a complete cytogenetic response. However, the addition of BMS-833923 did not seem to affect the potential for self-renewal as measured by colony forming culture assays, in contrast to *in vitro* study (70) and there was no observed clinical response in advanced CML patients or ALL patients. (NCT01218477) (104).

**Table 1:** List of clinical trials of SMO inhibitors in hematological malignancies (from clinical trials.gov).

Trial ID	Phase	Disease	HH agent	N	Outcome measure	Treatments	Outcomes	Publication or Status
NCT 953758	I	AML	Glasdegib	47	First cycle DLT	5, 10, 20, 40 and 80, 120, 180, 270, 400, 600 mg/day	DLT determined: MTD 400mg/day	(98)
NCT 2038777	I	AML	Glasdegib	49*	First cycle DLT	25, 50 and IOOmg/day	No DLT	(99)
NCT 1546038	Ib	AML	Glasdegib	52	MTD and RP2D	A: w/ LDAC, B: w/decitabine, C: w/ICT (induction chemo)	A+B: No DLT, C: Grade 4 polyneuropathy DLT. RP2D: IOOmg/day	(100)
NCT 3390296	lb/II	AML	Glasdegib	138'	AE and CRc	Drug combinations: PF-04518600, Avelumab, AZA, Utomilumab, GO, Glasdegib	None yet	Estimated completion date: December 29, 2024
NCT 1546038	II	AML	Glasdegib	71	CR w/ analysis defined as deaths in at least 40 of 60 patients >55yo	IOOmg/day w/ DNR and Ara-C	CR in 46.4% of 69 patients. In >55yo, 40.0%	(101)
NCT 1546038	II	AML	Glasdegib	132	OS	IOOmg/day w/ LDAC N=84 (or just LDAC N=41)	Median OS: w/o 4.9 mo, w/ 8.8mo	(102)
NCT 1841333	II	AML	Glasdegib	31	Relapse-free survival, Remission duration, OS, AE	After Autologous transplantation: IOOmg/d	Relapse free survival: 142 days (28–336), Remission duration: 333 (87–787) days, 28/31 experienced an AE, OS over I year was 20/31 (64.5%)	Completed April 2020
NCT04051996	II	AML	Glasdegib	46*	CR, OS, EFS	IOOmg/day after DAC for either 5 or 10 days	None yet	Terminated due to COVID-19 enrollment issues.
NCT 3226418	II	AML	Glasdegib	75*	CR, all patients >60yo	A: no Glasdegib; Ara-C and Idarubicin. B: Decitabine, Venetoclax, and Glasedegib	None yet	Est Comp: July 7, 2023
NCT 4093505	III	AML	Glasdegib	252*	MRD Negativity and EFS	2x2 study, 2 GO schedules; w/(o) Glasdegib 100mg/day	None yet	Estimated completion date: March 1, 2024
NCT 4168502	III	AML	Glasdegib	414*	MRD Negativity and DFS	After Autologous transplantation: Ara -C, DNR, and GO; w/(o) Glasdegib 100mg/d	None yet	Estimated completion date: October 1, 2026
NCT 03416179	III	AML	Glasdegib	720*	OS	Double Blind: w/ Ara-C and DNR	OS: Glasdegib 17.3 (15.2-18.5) months I Placebo 20.4 (17.6-NA) (too few events to give upper limit of 95% CI)	Completed January 24, 2022
NCT 03416179	III	AML	Glasdegib	720*	OS	Double Blind: w/ AZA	OS: Glasdegib 10.3 (7.7-12.4) months | Placebo 10.6 (8.4-13.3)	Completed January 24, 2022
NCT 02367456	Ib	AML and MDS	Glasdegib	73	CR and OS	Open label: 100mg/day w/ AZA	AML (n=30): CR in (6) 20.0%; OS at 6mo= (21) 70.0% MDS (n=30): CR in (5) 16.7%; OS at 6mo= 78.9%	(103)
NCT 01826214	II	AML or ALL	Sonidegib	70	OR	400 or 800 mg/d	Unclear if the data is not in yet or if only 1 patient (in the 400mg group) had a CRi and nothing else happened.	Completed May 2015
NCT04231851	II	AML w/ MDS	Glasdegib	30	EFS	100mg/day after CPX- 351 (Ara-C + DNR)	None yet	Estimated completion date: September 30, 2022
NCT 01944943	II	B-Cell Lymphoma or CLL	Vismodegib	31	OR, PFS, OS	150 mg/d	Terminated early due to lack of efficacy	Terminated October 2014
NCT 01218477	I	CML	BMS- 833923	33	DLT, RP2D, along with any major responses (MCyR and/or MHR)	0, 50, 100, 200mg 2/d for 7 days then 1/d (all w/ 100 or 140mg/d dasatinib)	Tolerable dose at 50mg/d, but little evidence of efficacy	(104)
NCT 01456676	I	CML	Sonidegib	11	MTD	400-800mg/d	No published data	Completed February 2014.
NCT 01842646	II	MDS	Glasdegib	35	Response Rate, OS, EFS, (2yr4mo)	(N=35) Glasdegib 100mg/day in refractory MDS patients	No CR, 5.7% HI, 64.5% SD, 30% PD; OS: 10.2 months; EFS: 6.4 months	(105)
NCT01371617	II	MF	IPI-926	14	OR	160, 130, or 110 mg/d	ORR<10%, some reduction of Gli1 levels, but overall was not considered to be a drug worth pursuing	(106)
NCT 02226172	II	MF	Glasdegib	21	Patients' w/ spleen size reduction, AE,	In patients previously treated with Ruxolitinib: Placebo or 100mg/d	In lead-in cohort: Drug was considered safe, but did not meet minimum efficacy requirements	Terminated April 2 2018
NCT 02593760	Ib	MF	Vismodegib	10	Patients' w/ spleen size reduction, AE, CR, safety if drug	150 mg/d and placebo, w/ Ruxolitinib	Completed, found Vismodegib to be safe but had no improvement over Ruxolitinib alone	(107)
NCT 02254551	II	MM	Sonidegib	7	MTD, Time to Progression, OR	400, 600, and 800 mg/d, with injections of Bortezomib	Safety requirements not met in the leadin, 400mg was deemed to be too great a toxicity	Terminated February 16, 2017
NCT 02086552	II	MM	Sonidegib	28	CR, OS, PFS	After SCT, 400mg/d w/ Lenalidomide	(n=26) CR: 8 (31%), VGPR: 11 (42%), PR: 6 (23%), SD: 1 (4%). Toxicity a problem, only 10 completed the treatment regiment	Completed
NCT 01330173	Ib	MM	Vismodegib	50	Change in MM CSC blood count	After SCT: daily, no given dose	No data	Completed November 2014
NCT 02129101	I/Ib	Myeloid Malignancies	Sonidegib	63	MTD and best overall response	0-400mg w/ AZA	MTD: 200mg/day, Response: 76% R/R was SD and OS was 7.6 mo	(108)
NCT 01787552	Ib/II	PMF	Sonidegib	50	First cycle DLT, spleen size reduction	400 mg/d + 5-20mg/d INC424	Ended early due to Novartis divesting sonidegib: (results have not been posted)	Ended April 10, 2018

The asterisk (*) indicates an estimated or not yet finalized enrollment number for the given clinical trial.

### Glasdegib phase I trials

Glasdegib is an oral, selective HH inhibitor that binds SMO (112) Clinical trials have been limited to the study of its effect of myeloid malignancies: Japanese phase I NCT 02038777 (99), US and Europe phase I NCT 00953758 (98) and a study in myelofibrosis (NCT 02226172)

The Japanese trial tested doses of 25, 50, and 100mg. In the AML group (n=7), there was 1 complete remission and four patients who achieved stable disease; in the MDS group there was one marrow complete remission and two patients who achieved stable disease. Further, GLI1 was found to be significantly downregulated in patients who were in the 50 or 100mg treatment group (99).

These results are mirrored in the American and Italian trial, which tested for doses ranging from 5 to 600mg. In the AML group (n=28), 16 showed some possible biological activity: 1 patient had complete remission, four had partial remission, four had a minor response, and seven achieved stable disease. 3 of 6 MDS patients achieved stable disease, with two of those showing hematological improvement, 2 of 7 myelofibrosis patients demonstrated some clinical improvement and the one enrolled CMML patient also achieved stable disease. One patient out of five with CML achieved a partial cytogenetic response (98). Martinelli et al. found a maximum tolerated dose of 400mg daily, however 100mg was suggested as the phase 2 dose based on tolerability and target inhibition (98).

### Bright Aml 1003

BRIGHT AML 1003 (NCT 01546038) examined the use of Glasdegib in various combinations for AML or high-risk MDS. The Phase Ib/II study, tested glasdegib 100mg in combination with standard “7+3” induction chemotherapy (ICT) for fit patients and glasdegib in combination with LDAC for unfit patients unable to tolerate high dose chemotherapy.

For fit patients, the 1003 study was a single arm, phase II of glasdegib 100mg daily plus ICT with CR rate as the primary endpoint (102). Although the trial failed to meet the prespecified CR rate, relapse rates and overall survival (OS) was significantly improved in risk groups when compared to expected outcomes by European LeukemiaNet category (113). This pattern of decreased relapse rate without an effect on CR is characteristic of a CSC targeting agent, as one would expect a drug targeting CSCs to not affect the bulk tumor, but rather to inhibit the surviving CSCs ability to self-renew and reconstitute the disease.

Patients ineligible for intensive induction therapy on the 1003 study were randomized to LDAC alone or LDAC in combination with glasdegib in an open label design. CR rates and survival at one year was significantly increased in the glasdegib combination, with acceptably low toxicity (102). FDA approval was made largely based upon these data (111). A major weakness of this trial, however, was the open label design which may have contributed to the significantly shorter treatment duration for LDAC alone patients and could confound the results. More importantly, extremely favorable response rates to venetoclax combinations with hypomethylating agents in similar, unfit AML patient populations have limited clinical enthusiasm for the LDAC+glasdegib combination.

### Glasdegib phase III and in-progress trials

There are currently three large phase III trials recently reported or in progress. BRIGHT AML 1019 (NCT 03416179) is a global randomized double-blinded placebo control trial of glasdegib in combination with standard therapy for front line AML. Fit patients eligible for induction chemotherapy are offered cytarabine and daunorubicin (7 + 3) plus glasdegib or placebo. Unfit patients are treated with azacitidine and glasdegib or placebo. Neither arm hit their primary overall survival endpoint. However, detailed subgroup analysis from this large phase III study has not yet been presented.

Two trials examine glasdegib in combination with gemtuzumab ozogamicin (GO), an antibody-drug conjugate that targets cells expressing CD33, a marker that is expressed in tumor cells in almost all AML patients (114). A German trial, GnG (NCT 04093505) is using a two-by-two factorial design with two different GO schedules followed by the use of 100mg per day glasdegib or a placebo. It aims to find the effect of the treatments on measurable residual disease (MRD) and event free survival (EFS) at 2 years. An Italian trial (NCT 04168502) is looking at younger patients and considers the use of glasdegib as maintenance after a patient receives an autologous or allogenic stem cell transplant (SCT). Patients receive 7 + 3 ICT (Ara-C and DNR), GO and a stem cell transplant, followed by 100mg glasdegib per day. The study will consider MRD following the SCT and GO treatments, and then measure disease free survival at 2 and 5 years.

## Discussion

While SMOi has seen some success in clinical study for AML, the processes of interpreting the results and charting future directions for the treatment are hindered by current gaps in our mechanistic understanding of the role of HH signaling in hematological malignancies. It remains unclear whether the tumor or the microenvironment are the primary targets for SMOi. Demonstrating modulation of HH signaling in tumor samples has been challenging and has prevented the development of useful pharmacodynamic biomarkers.

Based on data from both clinical and preclinical study, we suggest that signaling is active in stromal cells that support the hematopoietic and leukemic stem cell microenvironments. This hypothesis would explain the apparently contradictory results from earlier examinations of HH signaling in hematopoiesis, as well as the pattern of SMOi preventing relapse rather improvement of initial response in clinical trials. However, this hypothesis needs to be specifically tested by specifically knocking out Smo in the stroma without affecting its function in HSCs/CSCs. Establishing a greater understanding of the mechanisms of HH signaling in hematological malignancy will allow for better interpretation of its clinical benefits and improved implementation as a targeted therapy.

## Author contributions

Conception of idea was done by AM. Manuscript writing and figure preparation was done by TL. Manuscript editing was done by both authors. Both authors contributed to the article and approved the submitted version.

## Funding

This work was supported by a grant from National Heart, Lung and Blood Institute (NHLBI R01 R01HL138414) to AM.

## Acknowledgments

We would like to thank our colleagues in the Merchant Lab at the Cedars-Sinai Medical Center.

## Conflict of interest

AM has received research funding from Pfizer and served on an advisory board for Novartis.

TL declares that the research was conducted in the absence of any commercial or financial relationships that could be construed as a potential conflict of interest.

## Publisher’s note

All claims expressed in this article are solely those of the authors and do not necessarily represent those of their affiliated organizations, or those of the publisher, the editors and the reviewers. Any product that may be evaluated in this article, or claim that may be made by its manufacturer, is not guaranteed or endorsed by the publisher.
